# Influence of Distance from the Host on Parasitisation by *Psyttalia concolor* (Szépligeti) (Hymenoptera: Braconidae)

**DOI:** 10.3390/insects10100316

**Published:** 2019-09-25

**Authors:** Nuray Baser, Gabriella Lo Verde, Gülay Kaçar, Flutura Lamaj, Vincenzo Verrastro, Alberto Lombardo, Francesco Tortorici, Virgilio Caleca

**Affiliations:** 1 CIHEAM-IAMB—International Centre for Advanced Mediterranean Agronomic Studies, 70010 Bari, Italy; baser@iamb.it (N.B.); lamaj@iamb.it (F.L.); verrastro@iamb.it (V.V.); 2 Department of Agricultural, Food and Forest Sciences, University of Palermo, 90128 Palermo, Italy; francesco.trt@gmail.com (F.T.); virgilio.caleca@unipa.it (V.C.); 3 Natural Science Faculty Gölköy, Bolu Abant Baysal University Agricultural, 14280 Bolu, Turkey; gulaysahan@yahoo.com; 4 Department of Engineering, University of Palermo, 90128 Palermo, Italy; alberto.lombardo@unipa.it

**Keywords:** olive fruit fly, medfly, laboratory host, ovipositor length, parasitoid rearing

## Abstract

The olive fruit fly, *Bactrocera oleae*, is considered the main olive pest worldwide, and has been the target of biological control programmes through the release of the braconid parasitoid *Psyttalia concolor*. Laboratory tests were performed to evaluate the influence of distance from the host on parasitisation, placing larvae of the substitute host *Ceratitis capitata* at seven distances (0, 0.5, 1, 1.5, 2, 2.5, 3 mm) and four different time periods (7, 15, 30, 60 min). Moreover, field collected olives of Ogliarola Barese cultivar infested by *B. oleae* were exposed to *P. concolor* females to confirm its ability to parasitise *B. oleae* in small olives. *Psyttalia concolor* oviposition was inhibited at 2.5 and 3 mm due to the ovipositor length of the parasitoid females (2.7 mm). Hosts were easily parasitised at distances between 0 and 1.5 mm. The thin fruit pulp (up to 3.5 mm) of field collected olives allowed the parasitisation to occur also in mature fruits. At the best combination distance/time (0 mm, 30 min), tests performed with different larvae/parasitoid female ratio showed an increasing emergence of *P. concolor* (from 20% to 57%) with larvae/parasitoid ratio increasing from 0.11 to 0.74. The results of the present study might optimise the mass rearing of *P. concolor*, through a proper setting of its parameters, such as the host/parasitoid ratio, exposure distances, and interaction time.

## 1. Introduction

Olive cultivation is of remarkable economic importance in the Mediterranean area. Yield losses for harvested fruits due to the olive fruit fly were estimated to be at least 15% of the total olive production [1]. The olive fruit fly, *Bactrocera oleae* (Rossi) (Diptera: Tephritidae), is commonly considered the key pest of olive fruits in the Mediterranean climatic conditions, and the tolerable limit of *B. oleae* exit holes to obtain high quality oil has been recently assessed [2].

The common technique for pest control is the application of broad-spectrum insecticides on the whole canopy or spraying with poisoned-protein baits on part of the tree canopy. Moreover, repeated treatments cause resistance to synthetic and natural compounds [3,4,5]. Integrated pest management is considered the best strategy for sustainable crop protection. This includes the use of less susceptible plant varieties, cultural techniques, the use of natural enemies, microbial pesticides (entomopathogenic bacteria, viruses, and fungi), natural plant insecticides, semiochemicals, and insect growth regulators. Natural control by predation on eggs and larvae, which are protected inside olive fruits, is not effective in reducing the pest population level [6]. On the other hand, soil arthropods can cause substantial mortality on olive fruit fly pupae wintering in the soil [6,7,8].

In the Mediterranean basin, the solitary larval parasitoid *Psyttalia concolor* (Szépligeti) (Hymenoptera: Braconidae) has been used with patchy results for biological control of the olive fruit fly through inundative releases [9,10,11,12,13] or augmentative/inoculative releases [14,15,16,17,18,19,20,21,22,23]. Moreover, in California (USA), after the permanent establishment of olive fruit fly, the two sub-Saharan parasitoids *Psyttalia humilis* (Silvestri) and *Psyttalia lounsburyi* (Silvestri) have been released in the field [24,25].

The renewed interest in using parasitoids for the classical biological control of fruit fly encouraged studies aimed at improving the morphological description and taxonomic position of the parasitoids of *B. oleae*, in particular the genus *Psyttalia* Walker. Therefore, the taxonomic position of *P. concolor* has been recently revised using molecular analysis, assessing that it is present only in the Mediterranean Basin and *B. oleae* is its only natural host [26]. The same authors identified the species previously considered to be *P. concolor* in sub-Saharan areas as *P. humilis*, which is morphologically indistinguishable from *P. concolor* [26]. *Psyttalia concolor* is thought to have been introduced as a biological control agent in the Italian Peninsula [27], whereas it seems acceptable to consider the species as native to Sicily, Southern Sardinia, and Southern Calabria [2,28,29]. Currently, *P. concolor* is the most active parasitoid in cultivated olive areas of Sicily [30].

In order to optimize parasitoid rearing, several studies have been carried out in the past years, investigating the effect of different host/parasitoid ratios on the percentage of emerged *P. concolor* with 24 h of exposure time [31] or at different exposure intervals with a ratio of 2 hosts per parasitoid [32]. Further aspects of the parasitoid behavior have been also investigated, like the superparasitism and the discrimination ability of parasitized host [31,33,34,35] or the influence of olfactory cues and early adult learning on host preferences by the parasitoid females [36,37]. More recently, also *P. lounsburyi* and *P. humilis* have been evaluated for their use in the classical biological control of *B. oleae* in California. Some biological parameters have been investigated under laboratory conditions, among them developmental time, adult longevity, fecundity [24,38,39], whereas the effect of rearing parameters like the larval host stage or the exposure time on mass-rearing production of *P. lounsburyi* has been assessed, in order to develop a sustainable rearing technique of the parasitoid [40,41].

Among the factors affecting the parasitisation efficiency of *P. concolor*, fruit size seems to play a relevant role, as shown by field observations [42]. Besides, laboratory tests carried out using *P. humilis* (sub *P. concolor*) from Kenya and *P. lounsburyi* demonstrated that differences in parasitisation rates can be related to the length of the ovipositor and the pulp thickness of olives [43,44].

The aim of this laboratory study was to determine the effect on *P. concolor* production of both exposure time and distance between the parasitoid females and its host, in order to improve the parasitoid mass rearing.

The determination of optimal distance and exposure time can be useful to improve the mass rearing method of the parasitoid. Moreover, the determination of optimal distance might aid the evaluation of varieties on which this parasitoid can be more effective against *B. oleae* in biological control.

## 2. Materials and Methods

### 2.1. Insects

Insect rearing and experiments were both conducted under laboratory conditions (24 ± 1 °C, 14 light: 10 dark cycle, 64% RH) at the Mediterranean Agronomic Institute (C.I.H.E.A.M.) of Bari, Italy.

The laboratory tests were performed using the medfly, *Ceratitis capitata* (Wiedemann), as alternative host of *P. concolor*. The laboratory colony of medfly was initiated from infested organic apricot field samples in Bari Province (Apulia, Italy), and had been reared since 2000 according to the commonly adopted procedures as described by [20,45,46,47]. Since then, *C. capitata* individuals from field infested fruits have been periodically added to the rearing in order to avoid problems dealing with prolonged breeding. Medfly adults were reared in plexiglass cages (40 × 50 × 45 cm) provided with a medium consisting of protein bait (30 g), dry yeast (8.4 g), sugar (40 g), and water (40 mL), and wet paper to supply water. Approximately 8000 *C. capitata* adults were placed into cages with one side covered by a net of tulle on which females laid their eggs. Eggs were collected daily into a water reservoir located at the base of the cage to avoid desiccation and added to an artificial pabulum, prepared as described in [48] and consisting of a mix containing bran (96.8 g), sugar (64.8 g), dry yeast (9.07 g), citric acid monohydrate (2.4 g), and sodium benzoate (2 g) suspended in water (200 mL). Medfly eggs (0.25 mL suspended in 50 mL of water) were then placed in cups filled with 375 g of the above described pabulum and kept in growth chambers until hatching. Mature larvae (third instar) jumping from substrate were collected in underlying trays filled with water and reared until adult emergence in order to establish a new generation of the insect or used in the experiments.

*Psyttalia concolor* rearing started in 2005 at C.I.H.E.A.M., using parasitoids provided by the “Centro Regionale Agrario Sperimentale (CRAS)” in Cagliari, Italy. Periodically, field populations of the parasitoid were added to the breeding population to avoid the problems associated with multi-annual breeding. Such populations were obtained from rotten fruits of *Rosa canina* L. 1753 and *Citrus sinensis* (L.) (Osbeck), infested by medfly at the Botanical Garden of the University of Bari (Apulia, Italy).

*Psyttalia concolor* was reared following the methods described by [20]. Adult parasitoids were supplied with water and a mixture of honey and pollen as a food resource, inside plexiglass cages (40 × 50 × 45 cm). In order to obtain a new generation of the parasitoid, approximately 10,000 mature medfly larvae placed into a rectangular tray with a tulle tissue base were put for 15–20 min in contact with the upper sides of the cages containing 10-day-old adult parasitoids (12,000, 50% females) [45]. After 17–20 days, adults of parasitoid emerged, while the fraction of not parasitised larvae (about 25%) developed into pupae, producing after 10 days medfly adults used for its additional breeding. Parasitoids for each test have been collected from a sample of contemporaneously parasitised host larvae.

### 2.2. Ovipositor Length of Psyttalia concolor

**Figure 1:**
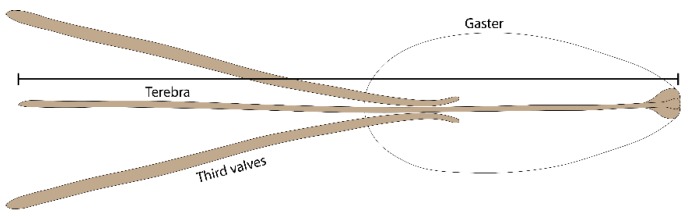
Schematic drawing of *P. concolor* gaster and ovipositor, and the adopted method for measuring terebra length.

To assess differences in the ovipositor length of *P. concolor* reared on *C. capitata* and *P. concolor* obtained from *B. oleae*, 23 females of *P. concolor* reared in the laboratory on *C. capitata*, and 47 females that emerged from *B. oleae* puparia obtained from cultivated olives (25 specimens) and Mediterranean wild olives (22) collected in 2010–2011 in Sicily (Trapani province) were dissected to extract the ovipositor from the gaster. The length of the terebra (first and second valvifers, from base to tip) was measured under a stereomicroscope using an ocular micrometre, following the methodology shown in Figure 1 [43].

### 2.3. Parasitisation Assays

**Figure 2:**
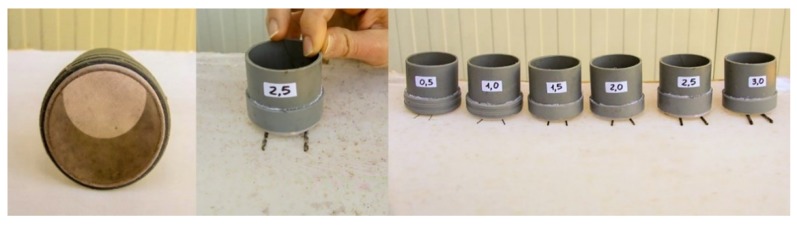
The plastic cylinders with a base made of tulle tissue used as containers for medfly larvae to be parasitised and the metal bars used to set the different distances in laboratory tests.

To evaluate the effect of the distance between *P. concolor* females and the medfly larvae on the level of parasitisation, parasitoids were located in plexiglass cages (40 × 40 × 40 cm). The roof of the cages was made of a well tensed tulle tissue, allowing oviposition by the adult female parasitoids. *Ceratitis capitata* mature larvae were placed into plastic cylinders (5 × 5 cm, height × width) with a base made of the same tulle material as the above described cages (Figure 2). For each replicate, approximately 1200 adult *P. concolor* females (10 days old) and approximately 130 *C. capitata* larvae were used (ratio between *C. capitata* larvae and *P. concolor* females 0.11).

The distances between the cages containing the adult *P. concolor* and the cylinders containing the medfly larvae were set at 0.5, 1, 1.5, 2, 2.5, and 3 mm by means of metal cylindrical bars (Figure 2) and the minimum distance was obtained by putting the cylinders in direct contact with the cage roof. The four tested exposure times were 7, 15, 30, and 60 min.

At the end of each test, the medlfly larvae were kept under the abovementioned laboratory conditions to obtain adults of both medfly and *P. concolor*. The number of *P. concolor* and *C. capitata* adults was recorded daily until 30 days after exposure to the parasitoid, then the number of dead pupae was also counted. For each distance/minute combination test, at least six replicates were performed.

Based on the results obtained in the above described trials, further tests were performed adopting the optimal distance and time with an increasing ratio of *C. capitata* larvae and *P. concolor* females (0.26, 0.64, and 0.74 *C. capitata* larvae/*P. concolor* females) to assess the effect of the ratio on the percentage of obtained parasitoids. Three replicates were performed for each further test.

### 2.4. Parasitisation Tests on Ogliarola Barese Olives

In order to confirm the ability of *P. concolor* to parasitise *B. oleae* in small olives, a two-year experiment was carried out in 2013–2014 using olives of the Ogliarola Barese cultivar. This cultivar, at harvest, produces an elliptical (l/w 1.30) small fruit (<2 g, transverse width 13 mm, maximum pulp thickness 3.5 mm [49]. Olive samples from organic olive orchards were collected at the Mediterranean Agronomic Institute of Bari from different trees. As confirmed by tests results, no *P. concolor* population was present in the study area. Samplings were carried out from the beginning of July until harvest time (17 and 14 samples in 2013 and 2014, respectively). Only fruits infested by *B. oleae* were chosen for the tests, selecting them by an external observation of oviposition punctures. From the selected olives, six groups of fifty-one were randomly formed. Three of these groups were separately exposed to 10-day-old *P. concolor* adults, whereas the remaining three groups were used as control. Each olive group was exposed to 10 females and 10 males of *P. concolor* for 11 days, to allow the development of *B. oleae* eggs and first instar larvae into third stage larvae, the targets of parasitisation. On each test date, olives and adult parasitoids were kept in cages provided with honey, a sugar mixture, and water. The control cages were arranged only with infested olives. All cages were kept at 24 °C and 64% RH and provided with 14 h of light daily. The number of *B. oleae* and *P. concolor* adults were counted 30 days after the end of the test. Finally, in 2014, the length and width of about 80 olives and their kernels were measured at four sampling dates, including the last one (first decade of November), in order to calculate their pulp thickness.

### 2.5. Data Analysis

The length of terebra of *P. concolor* emerged from the laboratory host *C. capitata*, from the natural host *B. oleae* collected from cultivated olive and from Mediterranean wild olive was compared by 1-way ANOVA, after a Box-Cox transformation of data and a normality check through Anderson Darling test (AD = 0.46, *p* = 0.25). The analysis was followed by Bonferroni post-hoc test.

For laboratory tests, a preliminary analysis was carried out to assess the effect of distance on the percentage of emerged *C. capitata* adults and on not emerged insects in the trials performed at 60 min, including the test performed in absence of parasitoid females. A binary logistic regression was applied. Distance was considered as a categorical factor, because the behavior of the odds ratio is not linear. The analysis has shown a significant effect of distances for both outputs (*C. capitata* and not emerged insects), comparing the confidence interval for each odds ratio. Afterwards, a multiple comparison approach was adopted, performing a Bonferroni correction to obtain the probability of each event percentage (*P. concolor* or *C. capitata* emergence, no insect emergence) and the confidence intervals with a 99% probability level, which leads to a familywise error rate near to 95%. Based on the results of this analysis, we decided to exclude the distances higher than 2 mm and the exposure time of 60 min from the subsequent analyses.

To assess the effect of the different exposition distance and time on the percentage of emerged *P. concolor*, *C. capitata* and not emerged insects, a binary logistic regression was performed. In this analysis the two factors, distance and time, considered as categorical for the previous reason, and their interaction were investigated. Because of the presence of interaction in the model, the odds ratio values and related confidence intervals (C.I.) are not available. To assess the significance of percentage of different levels, the percentages estimated according to the model, and the related C.I., were calculated. Also, in this case, a Bonferroni correction approach was adopted—therefore the individual confidence levels have been calculated as 99%, leading to a familywise error rate near to 95%.

Finally, the significant effect of the different host/parasitoid ratio (all at 0 mm distance and 30 min) on percentages of *P. concolor*, *C. capitata* and not emerged insects was assessed using a binary logistic regression. Differences among the different levels of host/parasitoid ratio were then assessed comparing the confidence interval for each odds ratio.

In all the binary logistic regression procedures, the Hosmer-Lemeshow goodness of fit test resulted as significant.

All statistical analyses were performed using MINITAB software (Minitab, Inc., State College, PA, USA).

## 3. Results

**Figure 3:**
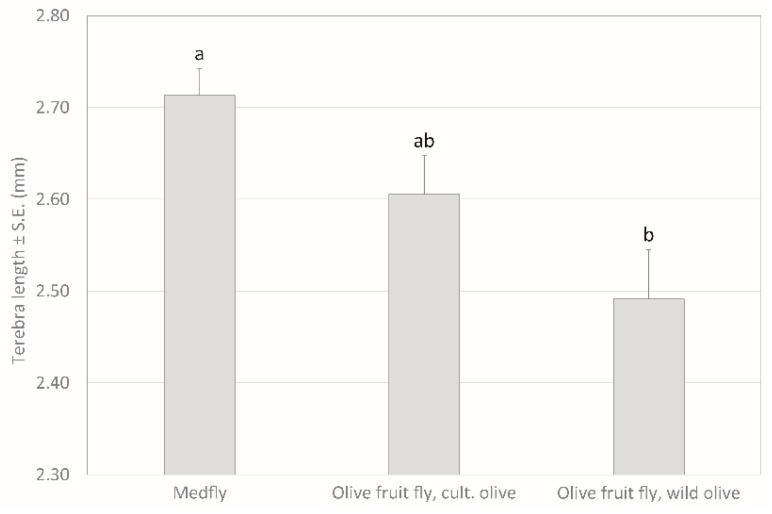
Differences in the length of terebra in *P. concolor* emerged from the laboratory host *C. capitata* and from the natural host *B. oleae* collected in Mediterranean cultivated and wild olive. Different letters indicate significant differences (1-way ANOVA followed by Bonferroni post-hoc test).

Significant differences in the terebra length were found between the *P. concolor* females emerged from the laboratory host *C. capitata* (2.71 mm) and from the natural host *B. oleae* collected in Mediterranean wild olive (2.49 mm, Figure 3). The length of terebra in *P. concolor* emerged from *B. oleae* collected in cultivated olive showed intermediate values (2.61 mm) and was not significantly different from the other two (1-way ANOVA followed by Bonferroni post-hoc test, F_2,62_ = 7.85; *p* = 0.001).

**Figure 4:**
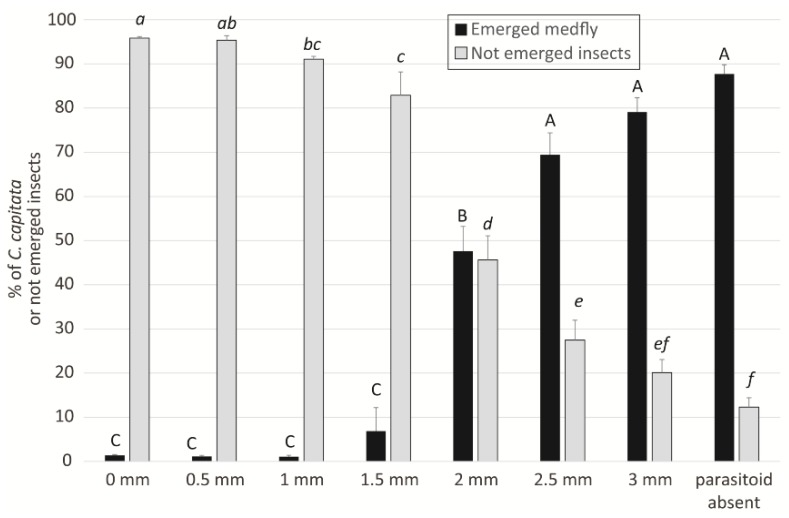
Comparisons of the percentages of emerged *C. capitata* adults (black bars) and of not emerged insects (grey bars) obtained at different distances from the host in the 60 min tests performed without the parasitoid or with the parasitoid. Different letters (capital for emerged medfly, italic for not emerged insects,) indicate significant differences within the same variable (Bonferroni multiple comparisons test).

Regarding tests performed with different combinations of exposure distance and time, the first step of the statistical analysis, limited to medfly emergence and not emerged insects in the 60 min tests compared to the control tests (without *P. concolor* females), revealed that no differences were present in the percentage of emerged medflies between the tests carried out without the parasitoid and those performed at 2.5 and 3 mm distances (F_7,56_ = 183.14; *p* < 0.01). The percentage of not emerged insects in the tests without the parasitoid and at 3 mm distance did not result statistically different, the latter being not different also from that recorded at 2.5 mm (F_7,61_ = 138.02; *p* < 0.01, Figure 4). This result is consistent with the terebra length (2.71 mm) of *P. concolor* reared on *C. capitata.* Moreover, the percentages of medfly emergence and of not emerged insects at 2 mm were statistically different from all the other distances, showing that also at 2 mm the parasitoid has difficulty in parasitising the host, probably because of the limited ovipositor length and of the difficult host recognition. For these reasons, we decided to exclude the distances from 2 mm on and the 60 min exposure time from the following analyses.

In tests with distances up to 1.5 mm and exposure time up to 30 min, the percentage of emerged *P. concolor* and *C. capitata,* and of not emerged insects was significantly affected by the two considered factors, distance and exposition time, and by the interaction between them (Table 1).

**Figure 5:**
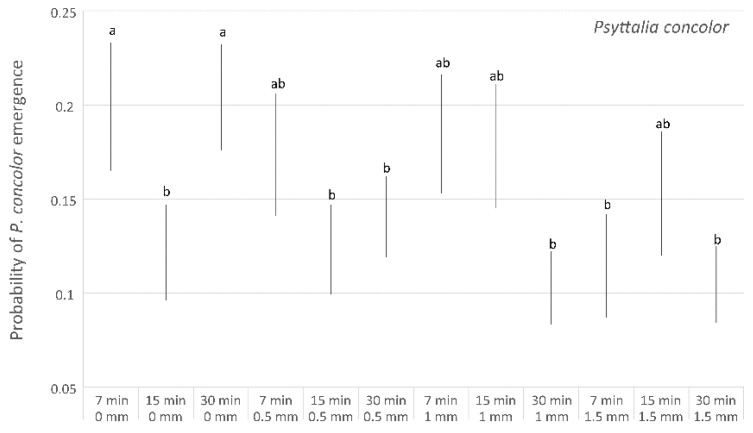
Differences in the probability of *P. concolor* adults emergence in tests performed with different exposition distances and time. Different letters indicate significant differences (Bonferroni multiple comparisons test).

**Figure 6:**
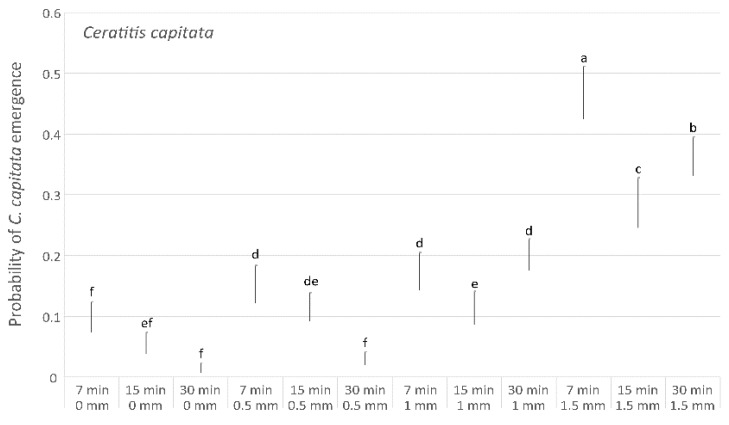
Differences in the probability of *C. capitata* adults emergence in tests performed with different exposition distances and time. Different letters indicate significant differences (Bonferroni multiple comparisons test).

**Figure 7:**
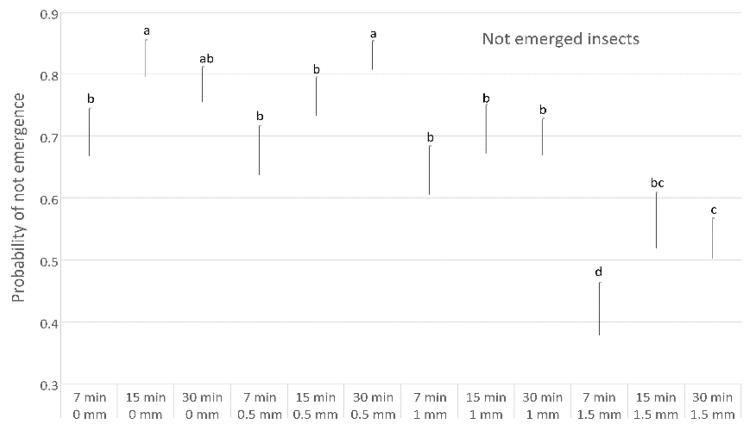
Differences in the probability of not emergence in tests performed with different exposition distances and time. Different letters indicate significant differences (Bonferroni multiple comparisons test).

In particular, up to the distance of 1.5 mm the parasitoid oviposition seems to be not affected by distance, as shown by the probability of *P. concolor* (Figure 5) and *C. capitata* emergence (Figure 6) and by the probability of not emergence (Figure 7). This latter result could be due to high superparasitism performed by *P. concolor* at 0–1.5 mm distances, as shown also by the low emergence of both *P. concolor* and *C. capitata*.

The maximum percentage of emerged *P. concolor* (20.2%) was obtained at 0 mm and with 30 min of exposure.

**Figure 8:**
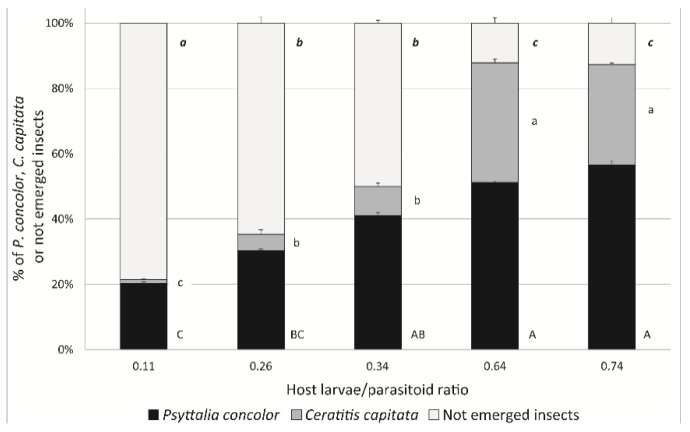
Percentages of emerged *P. concolor* and *C. capitata* adults and of not emerged insects in tests performed at 0 mm for 30 min and with different host larvae/parasitoid ratios. Different letters indicate significant differences.

Finally, the effect of the larvae/parasitoid ratio on the percentage of obtained parasitoids, in the tests performed at a distance of 0 mm and with a duration of exposure of 30 min, resulted always significant (*P. concolor*: Chi-Square_4,19_ = 119.13, *p* < 0.01; *C. capitata*: Chi-Square_4,19_ = 263.57, *p* < 0.01; not emerged insects: Chi-Square_4,19_ = 420.13, *p* < 0.01). The highest parasitoid percentages (56.6% and 50.9%) were recorded for the two highest larvae/parasitoid tested ratio, 0.74 and 0.64, respectively (Figure 8). In contrast, the percentage of not emerged insects decreased with the increasing larvae/parasitoid ratio and was, significantly, highest with the lowest larvae/parasitoid tested ratio (0.11). Moreover, both *P. concolor* and *C. capitata* percentages were significantly lower at 0.11 larvae/parasitoid ratio in comparison with all other tested ratios, demonstrating excessive superparasitism.

### Parasitisation Tests on Ogliarola Barese Olive

**Figure 9:**
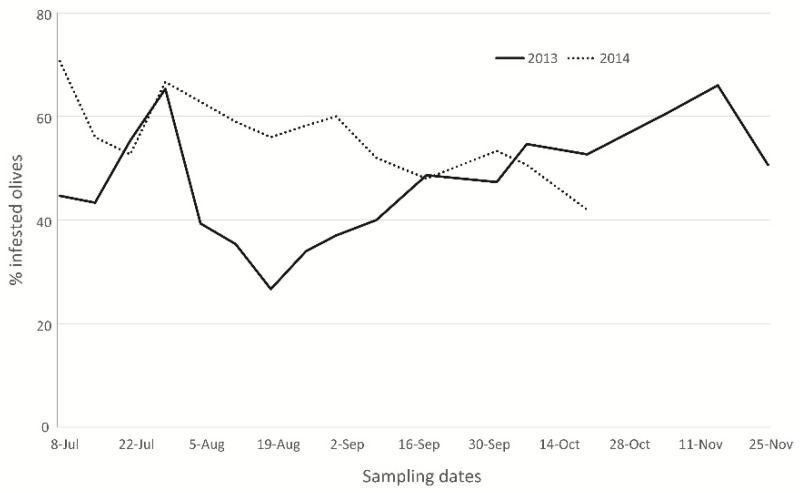
Percentage of *B. oleae* adults emerged from olives not exposed to *P. concolor* parasitisation.

From olives that were not exposed to *P. concolor,* only *B. oleae* adults emerged, confirming the absence of the parasitoid in the sampled olive orchard. The average infestation level (number of olive fruit flies emerged from 100 drupes) recorded on the different dates for both years was quite variable, ranging from 27% to 71% (Figure 9).

**Figure 10:**
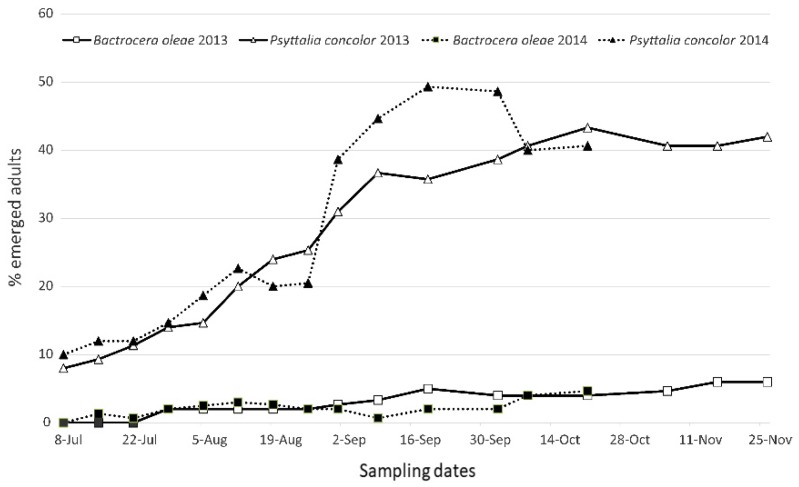
Percentage of *P. concolor* and *B. oleae* adults emerged from infested olives collected in the field at different sampling dates and then exposed to *P. concolor* parasitisation.

From olives exposed to *P. concolor,* the parasitoid adults emerged from each olive replicate, whereas only a few *B. oleae* adults emerged (Figure 10), demonstrating that the parasitoid females reached the suitable host larvae inside olives and that the pulp thickness of the sampled Ogliarola Barese olives, which reached the highest mean value of 2.5 mm in the sample collected in November, did not limit its parasitisation. The lower number of *P. concolor* that emerged in the first part of the season in both years could be due to the low level of third instar *B. oleae* larvae, which is the preferred stage for parasitisation.

## 4. Discussion

In solitary parasitoid species, the size of the host can influence both the parasitoid sex ratio [50] and size [51]. The parasitisation of different host species regards generalist parasitoids, but also the oligophagous parasitoid species that can be reared on alternative host species for their use in biological control programmes. This is the case of *P. humilis, P. lounsburyi* and *P. concolor,* reared on *C. capitata* mature larvae in order to be released in olive orchards to limit *B. oleae* populations. Females of *P. humilis* showed differences in the terebra length when emerged from the two hosts *C. capitata* (2.93 mm) and *B. oleae* (2.48 mm), both reared in the laboratory [44]. In our study, we provide the first measurement of *P. concolor* terebra, because in a recent paper [44], where insects used for terebra measurements were attributed to *P. concolor,* the correct reared species was *P. humilis,* based on results provided in [28]. Significant differences in the ovipositor size were found between parasitoids obtained from laboratory reared *C. capitata* compared with *B. oleae* grown in wild olives, whereas the parasitoids emerged from *B. oleae* developed in cultivated olives showed intermediate values of ovipositor length. Moreover, differences in the ovipositor length recorded for *P. concolor* are consistent with the results obtained for *P. humilis* [43] and confirm the high morphological similarity between the two species.

The ovipositor length of *P. concolor* females can be considered indicative of body size, as found in *P. humilis,* for which terebra length has been demonstrated to be related to forewing, hind tibia and total body length [43]. Thus, differences in the size of *P. concolor* could be due to the different size of parasitized *B. oleae* instars grown inside large cultivated or small wild olives, as fruits of different size provide a different thickness and total amount of pulp and nutrients for larval growth, as demonstrated in several commercially important olive varieties in California, which significantly influenced the weight of olive fly pupae [52].

Terebra length of reared *P. concolor* was demonstrated to play an important role in the parasitisation of *C. capitata* under laboratory conditions, because at the longest distances imposed between the parasitoids and the host (2.5–3 mm), *P. concolor* oviposition was almost completely inhibited. At a distance of 2 mm, parasitoid females still had some difficulty in reaching the host, whereas when the distances were set between 0 and 1.5 mm, the hosts were easily parasitised. Moreover, in our study the only host instar exposed to parasitoid females was the third instar larva, which was demonstrated, under laboratory conditions, to be the preferred instar for parasitism by *P. concolor* [34]. A laboratory study in which fully-grown *C. capitata* larvae were exposed to *P. concolor* parasitisation, showed that mobile hosts stimulated the ovipositor-probing behaviour more than immobilized hosts, indicating that host movement plays an important role in host location [53]. On this basis, it has been supposed that differences in the vibrations produced during feeding and/or movement by the second and third host instars could affect the host location efficiency by the parasitoid females [54]. Moreover, in a laboratory experiment, a significantly lower host location percentage and longer latency period were recorded for the second *C. capitata* larvae [34,54], leading to suppose that other factors, but not distance, influenced the host location. On the other hand, the establishment in California of *P. lounsburyi,* with a shorter ovipositor compared with *P. humilis,* but more specific on *B. oleae* [24,25,38,39], suggests that ovipositor length is not the only essential factor for successful parasitisation. Moreover, it has been observed that parasitoids with short ovipositors can parasitise *B. oleae* larvae in large olives with a high host density and a consequent pulp reduction, or when the mature host larvae move closer to fruit epidermis and prepares a thinner small area that will be pierced during the exit from the fruit [43].

In tests performed at the same distance, exposure time showed a positive effect on parasitisation rate, as well as the interaction between the two parameters distance and time. Moreover, shorter distances and longer times resulted in a higher superparasitism. The superparasitism in *P. concolor* rearings on *C. capitata* was investigated in previous studies [39,41], in which it was found that a superparasitism level up to two eggs per host larva could be considered useful to reach high percentages of parasitoid emergence. Moreover, it has been demonstrated that, under laboratory conditions, the parasitoid females show an innate ability to discriminate between larvae parasitised twice or only once, and that they prefer the latter, thus optimizing their oviposition decisions by deliberately avoiding superparasitised hosts [35].

The host/parasitoid ratio tested with the best distance/time combination obtained in the first tests (0 mm and 30 min), influenced both superparasitism and the percentage of emerged *P. concolor*. In particular, at 0.11 host/parasitoid ratio, a high percentage of not emerged insects was recorded, leading to suppose a high superparasitism level. On the other hand, at 0.64 and 0.74 host/parasitoid ratios, the percentage of not emerged insects was minimized, leading to obtain also *C. capitata* adults that could be used in the subsequent procedures for medfly rearing. In previous studies, the effect of different host/parasitoid ratios on the percentage of emerged *P. concolor* was investigated with 24 h of exposure time [40] and at different exposure intervals with a ratio of two hosts per parasitoid [41]. In the first case, the highest percentage of *P. concolor* was obtained with a ratio host/parasitoid 1:1 [40], whereas in the second study the highest percentage of parasitoids was obtained with a time exposure of 40 min [41]. Results obtained in our study, in which both factors were tested simultaneously, seems to be consistent with the previous studies, as the highest *P. concolor* percentage was reached in the test performed with an exposure time of 30 min and at 0.74 ratio host/parasitoid.

The distance effect found in the laboratory tests was confirmed by the infestation levels recorded for infested olives belonging to the Ogliarola Barese cultivar, where the thin fruit pulp allowed parasitisation also in mature fruits. The results obtained in tests performed using infested olives are consistent with two studies on *P. humilis* (sub *P. concolor*), [43] and *P. lounsburyi* [44], which reported that the two braconids more effectively parasitise the *B. oleae* larvae in smaller than in larger olive fruits. In our trials, the lower number of *P. concolor* that emerged in the first part of the season in both years could be due to the high level of young *B. oleae* larvae. Indeed, *P. concolor* has been demonstrated to clearly prefer to parasitise the third instar larvae, but also to successfully parasitise the second instar larvae of *B. oleae* on infested olive fruits [33]. The results obtained from tests carried out on field collected olives suggest that releases of *P. concolor* to control *B. oleae* could be useful only when applied early in the infestation season, when the olives’ pulp is thinner than 2.5–3 mm, or on cultivars with thin pulp, as host larvae suitable for parasitisation are usually located close to the olive kernel.

## 5. Conclusions

The present study confirms the importance of parasitoid distance from the potential host, both in laboratory conditions, and in olive cultivars with a thicker pulp [43,44] compared to the thin pulp of wild olives. Nevertheless, the host specificity of the parasitoid seems to play a more important role, as shown by the results of *P. humilis* and *P. lounsburyi* introduction into Californian olive groves, where only the latter, characterized by a shorter ovipositor but considered more specific on *B. oleae*, established [24,25,38,39].

The results of the study might optimise the mass rearing of *P. concolor*, through a proper setting of its parameters, such as the host/parasitoid ratio, exposure distances, and interaction time.

## Figures and Tables

**Table 1:** The binary logistic regression showed that percentages of emerged *P. concolor, C. capitata* and of not emerged insects are significantly influenced by the two factors, distance and exposition time, and by the interaction between them (in all the analyses the Hosmer–Lemeshow goodness of fit test was not significant).

*Psyttalia concolor*	DF	Adj Dev	Adj Mean	Chi-Square	p-Value
Regression	11	144.65	13.15	144.65	<0.001
Distance	3	28.78	9.593	28.78	<0.001
Minutes	2	35.14	17.571	35.14	<0.001
Distance ∗ Minutes	6	81.55	13.592	81.55	<0.001
Error	99	716.56	7.238		
Total	110	861.21			
***Ceratitis capitata***					
Regression	11	1767.29	160.663	1767.29	<0.001
Distance	3	404.78	134.928	404.78	<0.001
Minutes	2	88.64	44.318	88.64	<0.001
Distance ∗ Minutes	6	222.67	37.112	222.67	<0.001
Error	99	864.42	8.732		
Total	110	2631.71			
***Not emerged***					
Regression	11	881.37	80.125	881.37	<0.001
Distance	3	190.46	63.486	190.46	<0.001
Minutes	2	40.8	20.398	40.8	<0.001
Distance ∗ Minutes	6	41.69	6.948	41.69	<0.001
Error	99	496.25	5.013		
Total	110	1377.62			
